# A retrospective study about lymphangioma of the mesentery: An unusual cause of bowel volvulus

**DOI:** 10.1097/MD.0000000000046864

**Published:** 2025-12-26

**Authors:** Jian Sun, Chao Yang

**Affiliations:** a Department of Surgical Oncology, National Clinical Research Center for Child Health and Disorders, Ministry of Education Key Laboratory of Child Development and Disorders, China International Science and Technology Cooperation Base of Child Development and Critical Disorders, Chongqing Key Laboratory of Pediatric Metabolism and Inflammatory Diseases, Children’s Hospital of Chongqing Medical University, Chongqing, Yuzhong, China.

**Keywords:** abdominal pain, Mesenteric lymphangioma, pediatric, volvulus

## Abstract

Mesenteric lymphangioma is a rare benign lesion, and volvulus secondary to mesenteric lymphangioma is even less common, yet it can lead to severe complications. We retrospectively analyzed the clinical characteristics of pediatric patients with volvulus caused by mesenteric lymphangioma. Between January 1994 to August 2024, 28 pediatric patients were diagnosed with bowel volvulus attributable to mesenteric lymphangioma. Age, sex, clinical manifestations, ultrasound and computed tomography (CT) findings, operative findings, treatment, complications, and follow-up outcomes were reviewed retrospectively. The cohort comprised 13 boys and 15 girls, with 71.4% being preschool-aged. Most patients (21/28, 75%) sought medical attention within 24 hours of symptom onset. Clinical signs and symptoms were nonspecific and resembled those of intestinal obstruction. Ultrasound detected mesenteric lymphangioma in 26 patients, and CT identified it in all 28 patients; the “whirlpool sign” was observed in 24 and 27 patients, respectively. All patients underwent emergency laparotomy. Intestinal necrosis occurred in 5 cases and was associated with longer consultation delays (*P* = .008) and higher degrees of rotation (*P* = .008). Surgical procedures included resection of the affected intestinal segment with end-to-end anastomosis (20 patients), tumor enucleation (6 patients), and marsupialization with endometrial cauterization 2 patients. Postoperative complications consisted of bowel obstruction and incision infection in 2 patients. No recurrences were noted during follow-up. Volvulus secondary to mesenteric lymphangioma is a rare condition that presents with nonspecific clinical features. It should be considered in the differential diagnosis of acute abdominal pain in children.

## 1. Introduction

Lymphangiomas are benign tumors that arise from vascular and lymphatic tissues and most commonly involve the head and neck of children under 2 years of age.^[1]^ Mesenteric involvement of the small intestine is exceedingly rare, accounting for <1% of all lymphangiomas.^[2]^ Mesenteric cystic lymphangioma (MCL) is a benign tumor that originates from the lymphatic system. MCL is rare, with an incidence of approximately 1 in 250,000. This tumor is more common in pediatric patients, with 60% of cases appearing at birth and 40% by the age of 1. MCL accounts for 5% to 6% of benign tumors in children. These tumors are predominantly found in specific areas: 70% in the head and neck, 20% in the axillary region, and 10% in internal organs. Symptoms can appear with various clinical features ranging from asymptomatic abdominal mass to acute abdomen. Diagnosis is confirmed through histopathological findings. The main treatment for MCL is surgical resection, which can be performed via open exploratory laparotomy or laparoscopic techniques. In some instances, if the MCL is closely attached to the intestinal wall, an intestinal resection may be necessary.^[3]^ These lymphangiomas are usually asymptomatic and are often discovered incidentally. However, they can enlarge and compress adjacent structures, leading to bowel obstruction and ischemia. Rotation of the small intestine around a mesenteric lymphangioma may precipitate small-bowel volvulus (SBV), a rare but life-threatening surgical emergency. Prompt diagnosis is critical to prevent bowel necrosis and perforation, yet preoperative identification is difficult; definitive diagnosis is typically made during laparotomy or at autopsy.^[4]^ To better define the clinical characteristics of children with SBV secondary to mesenteric lymphangioma, we retrospectively reviewed all surgically treated cases at our institution over the past 3 decades.

## 2. Materials and methods

Approved by the ethics committee of Chongqing Medical University (CHCMU), this retrospective study included 28 children diagnosed with SBV secondary to mesenteric lymphangioma between January 1994 and August 2024. Each patient underwent emergency color Doppler ultrasonography (CDUS), abdominal radiography, and computed tomography (CT); all images were interpreted by experienced pediatric radiologists. Medical records were reviewed for age, symptom duration before presentation, major symptoms, precipitating factors, and physical-examination findings. Laboratory and imaging data comprised complete blood count (CBC), serum electrolyte levels, CDUS, and contrast-enhanced CT results.

All patients underwent emergent laparotomy. Depending on the lesion’s location and bowel viability after derotation, surgical procedures included segmental resection with end-to-end anastomosis, marsupialization with intimal cauterization, or tumor excision. Postoperative complications and follow-up outcomes were analyzed retrospectively.

Statistical analyses were performed with SPSS software, version 26.0 (SPSS Inc., Chicago). Categorical variables are presented as counts and percentages, and group differences were assessed with the χ^2^ test or Fisher exact test, as appropriate. Two-sided *P*-value of <.05 were considered statistically significant.

## 3. Results

### 3.1. Patient characteristics

From 1994 to 2024, 28 children with mesenteric lymphangioma–associated SBV underwent surgery at our institution. The cohort comprised 15 girls and 13 boys, with a mean age of 5.5 ± 3.0 years (range, 1.5–14 years); 71.4% were preschool age. No patient had a family history of the condition. A history of trauma or vigorous physical activity was recorded in 4 patients (14.3%).

### 3.2. Clinical signs

Detailed clinical, laboratory, and radiologic findings are summarized in Table 1. Most patients (21/28, 75%) sought medical attention within 24 hours of symptom onset; however, 2 presented more than 1 week after symptoms began. Both were ‘left-behind children – rural children cared for by grandparents while their parents worked in urban areas. The most delayed case involved an 8-year-old boy who had experienced recurrent abdominal pain for 4 months and chronic constipation.

**Table 1:** Clinical Findings of mesenteric lymphangioma accompanied with volvulus.

	N (%)		N (%)
Gender	–	History of trauma or activities	4 (14.3)
Male	13 (46.4)	Abdominal pain	28 (100)
Female	15 (53.6)	Emesis	24 (85.7)
Age (yr)	–	Nausea	22 (78.6)
0–3	11 (39.3)	Constipation	10 (35.7)
4–6	9 (32.1)	Fever	4 (14.3)
7–10	6 (21.4)	Bleeding per rectum	2 (7.1)
11–18	2 (7.2)	Abdominal distention	20 (71.4)
Duration of symptoms (d)	–	Abdominal tenderness	22 (78.6)
<1	21 (75)	Palpable mass	17 (60.7)
1–7	5 (17.8)	Weak bowel sound	14 (50)
7–14	1 (3.6)	High-pitched bowel sound	14 (50)
>14	1 (3.6)	Peritonitis	10 (35.7)

Abdominal pain was present in all cases (28/28, 100%), whereas vomiting and nausea occurred in 24/28 (85.7%) and 22/28 (78.6%) patients, respectively. Additional symptoms included constipation, fever, and rectal bleeding. Physical examination typically revealed nonspecific findings such as abdominal distention and tenderness; a palpable abdominal mass was detected in 17 patients (60.7%).

### 3.3. Laboratory and radiologic studies

Table 2 summarizes the key biochemical and radiologic findings. Electrolyte disturbances were present in 20 patients, most commonly hyponatremia, hypokalemia, and hypochloremia. More than half of the cohort exhibited neutrophilia, and 3 patients had hypoalbuminemia; all 3 had a prolonged history of chronic abdominal pain and vomiting.

**Table 2:** Laboratory and radiologic results of mesenteric lymphangioma accompanied with volvulus.

	N (%)		N (%)
Raised ratio of neutrophils	19 (67.9)	Abdominal plain film	–
Electrolyte disorder	20 (71.4)	Bowel obstruction	24 (85.7)
CDUS	–	Dynamic change	4 (14.3)
Cystic multiloculated mass	26 (92.9)	Abdominal CT	–
Whirlpool sign	24 (85.7)	Intraperitoneal cyst	28 (100)
Inconclusive owing to intestinal distension	1 (3.6)	Whirlpool sign	27 (96.4)
Misdiagnosed as mesenteric fat	1 (3.6)	–	–

CDUS = color Doppler ultrasound, CT = computed tomography.

Cystic multiloculated masses were found in 26 out of 28 lymphangioma cases (92.9%) using CDUS. Image quality was limited by intestinal distention in 1 patient, and another study was misinterpreted as mesenteric fat. The characteristic “whirlpool sign” was visualized in 24 patients.

**Figure 1.**
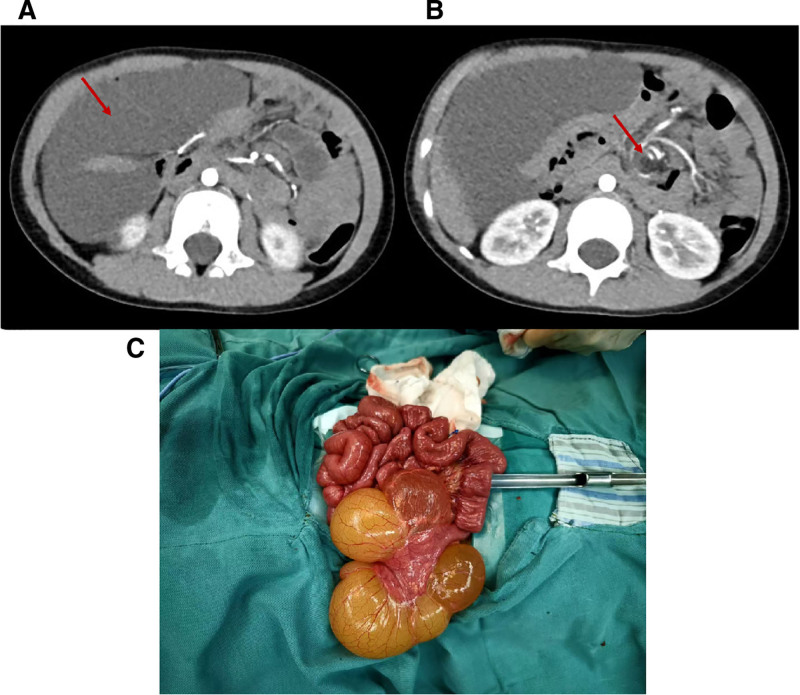
A patient with mesenteric lymphangioma accompanied by SBV. (A) A contrast-enhanced abdominal CT scan revealed a cystic, space-occupying lesion. (B) The CT image demonstrated a whirlpool sign, indicative of the superior mesenteric vein wrapping around the superior mesenteric artery. (C) Post-devolvulation, the lesion connected to the mesentery was excised, with resection performed on the anal side, 30 cm distal to the Treitz ligament. CT = computed tomography, SBV = small-bowel volvulus.

Radiologic evaluation also included abdominal radiography and contrast-enhanced CT. Plain films demonstrated features of bowel obstruction in 24 patients. Contrast-enhanced CT revealed thin-walled cystic lesions with enhancing walls and internal septations in all 28 patients. The whirlpool sign was detected in 27 cases (Fig. 1); the remaining patients showed an atypical relationship between the superior mesenteric artery and vein without a clear whirlpool configuration.

### 3.4. Operative findings and pathology

After laboratory and imaging evaluation, all patients underwent emergency laparotomy (Table 3). Among the 21 children who presented within 24 hours of symptom onset, only 1 (4.8%) had intestinal necrosis, whereas 4 of the 7 children (57.1%) who sought care after ≥24 hours exhibited necrosis (*P* = .008). Fourteen tumors were located in the jejunal mesentery, 12 in the ileal mesentery, and 2 in the duodenal mesentery. Lesion size averaged 8.5 cm (range, 3–15 cm). Clockwise rotation occurred in 17 patients (60.7%). A 360° twist in SBV was noted in 16 cases, 720° in 7, and 180° in 5; intestinal necrosis developed in 4 of the 7 children with 720° rotation.

**Table 3:** Surgical results of mesenteric lymphangioma accompanied with volvulus.

	N (%)		N (%)
Mass location	–	Rotation direction	–
Duodenum	2 (7.2)	Clockwise	17 (60.7)
Jejunum	14 (50)	Counter clockwise	11 (39.3)
Ileum	12 (42.8)	Treatment	
Rotation degree	–	Derotation + intestinal resection and anastomosis	20 (71.4)
180°	5 (17.9)	Derotation + enucleation of lymphangioma	6 (21.4)
360°	16 (57.1)	Derotation + lymphangioma partial resection and drainage	2 (7.2)
720°	7 (25)	–	–

The incidence of intestinal necrosis did not differ significantly with respect to rotation direction, presence of peritonitis, bowel-sound status, mass location, or neutrophil ratio (all *P* > .05). By contrast, a longer interval from symptom onset to presentation and a greater degree of rotation were both associated with necrosis (*P* = .008; Table 4).

**Table 4:** Statistical analysis of clinical characteristics between SBV with and without the necrotic intestine.

	SBV with the necrotic intestine (n = 5)	SBV without the necrotic intestine (n = 23)	*P* (Fisher exact test probabilities)
Direction			
Clockwise	3	14	.671
Counter clockwise	2	9	
Disease course			
<24 h	1	20	.008
More than 24 h	4	3	
Rotation degree			
<360°	1	20	.008
≥360°	4	3	
Sign			
Peritonitis	3	7	.249
No-Peritonitis	2	16	
Bowel sounds			
Weak bowel sound	1	13	.163
High-pitched bowel sound	4	10	
Mass location			
Duodenum	0	2	.753
Jejunum	3	11	
Ileum	2	10	
Ratio of neutrophils			
Raised ratio of neutrophils	4	15	.857
Normal ratio of neutrophils	1	8	

SBV = small-bowel volvulus.

Surgical management consisted of segmental resection with primary end-to-end anastomosis in 20 patients, tumor enucleation in 6, and partial excision with drainage in 2 cases in which the lesion was rooted at the mesenteric base and demonstrated intratumoral hemorrhage. Histopathologic examination confirmed the presence of lymphangioma in all specimens.

### 3.5. Postoperative care and complications

All patients who underwent enterectomy with end-to-end anastomosis received fasting and total parenteral nutrition for 3–5 days; enteral feeding was introduced once bowel function returned. Postoperative recovery was uneventful in 26 patients, with a median hospital stay of 6 days (range, 415 days. One patient developed an adhesive small-bowel obstruction that resolved with conservative management within 5 days. Another experienced an incisional wound infection on postoperative day 3, which responded to a 7-day course of antibiotics. No postoperative bleeding, anastomotic leakage, or intra-abdominal infection was observed.

### 3.6. Follow-up

All patients were followed long term, with a median duration of 65 months (range, 6–130 months). Each child underwent annual CDUS, and no clinical or sonographic evidence of recurrence was detected during follow-up.

## 4. Discussion

The etiology of mesenteric lymphangioma is not yet fully understood. It is most often suggested that these result from congenital malformations due to impaired lymphatic system development during embryogenesis or lymphatic obstruction caused by various pathological factors.^[5]^ Some investigators have suggested that mesenteric lymphangioma may be acquired secondary to chronic intermittent volvulus associated with malrotation^[6]^; however, none of our patients exhibited malrotation.

Two principal theories explain the link between SBV and lymphangioma. The first posits that the soft, pliant nature of mesenteric lymphangiomas makes them prone to torsion caused by changes in body position or abnormal peristalsis, thereby precipitating SBV. The second hypothesis proposes that persistent or intermittent volvulus obstructs lymphatic flow, leading to cyst formation; in such cases, the resulting lesion is typically a unilocular cyst without internal septations.^[7–9]^ In our series, every mesenteric lymphangioma was multilocular, suggesting that the lymphangioma was the primary lesion and that SBV developed secondarily.

Mesenteric lymphangioma typically manifests with nonspecific symptoms, the most common of which is abdominal pain.^[10]^ Additional findings often include a palpable abdominal mass and abdominal distention.^[11]^ Less frequent presentations comprise anorexia, weight loss, constipation, and a sensation of postprandial fullness. Intralesional hemorrhage can precipitate acute, severe pain with rapid mass enlargement. Large lesions have also been reported to cause intestinal obstruction, volvulus, and even acute pancreatitis.^[12,13]^

Volvulus secondary to a mesenteric tumor is rare in children. However, it can produce a closed-loop obstruction, leading to venous and arterial compromise, intestinal ischemia, and necrosis, with subsequent perforation and peritonitis. In our cohort, abdominal pain, vomiting, and nausea occurred in 28 (100%), 24 (85.7%), and 22 (78.6%) patients, respectively. A palpable mass was detected in 17 of 28 patients (60.7%). Because the clinical presentation is vague and no single finding reliably indicates volvulus, these symptoms may be mistaken for other acute abdominal conditions. Fever and mucoid bloody diarrhea, for example, can reflect bowel inflammation secondary to volvulus or an unrelated infectious enteritis. Intestinal ischemia is often characterized by pain that is disproportionate to physical findings.^[14]^ Assessing pain severity is challenging, and physical examination may be unreliable in young children who are frequently uncooperative, highlighting the importance of adjunctive investigations for accurate diagnosis.

Diagnosis of mesenteric lymphangioma with volvulus depends primarily on imaging. Plain radiographs typically reveal nonspecific signs of bowel obstruction, including dilated loops. Ultrasonography can identify multiloculated cystic mesenteric masses, but these may be misinterpreted as mesenteric fat owing to the mesentery’s fat-rich composition or may be obscured by intestinal distention. In our series, sonography was indeterminate in 1 patient due to gaseous distension and was misinterpreted as mesenteric fat in another. The “whirlpool sign” on gray-scale and color Doppler imaging – representing clockwise twisting of the superior mesenteric vein and mesentery around the superior mesenteric artery – is pathognomonic for SBV.^[15]^ This finding directly reflects the anatomic change produced by midgut volvulus. The positivity of sonography in our study was 85.7%, consistent with previous reports.^[16,17]^

CT is considered more reliable for definitive diagnosis, as it depicts a whirl-like configuration of the small-bowel loops (Fig. 1) and demonstrates higher sensitivity than ultrasound. In a prospective study by Zalcman et al, helical CT achieved 96% sensitivity and a 99% negative predictive value to diagnose or rule out intestinal ischemia in the context of acute small-bowel obstruction.^[18]^ Heightened clinical awareness is therefore essential. In children with chronic or intermittent abdominal pain and partial bowel obstruction – especially those with a palpable abdominal mass – mesenteric lymphangioma with volvulus should be strongly suspected. Ultrasound and CT are effective in detecting both the mass and the whirlpool sign and are thus recommended as the imaging modalities of choice.^[19–22]^

Emergent laparotomy is recommended for mesenteric tumors complicated by volvulus. After derotation, management depends on tumor type, lesion location, and bowel viability. The primary intraoperative goal is complete tumor excision while preserving mesenteric vessels. Well-circumscribed lesions can usually be managed with simple enucleation. When the tumor is densely adherent to the mesenteric border of the small intestine, segmental resection with end-to-end anastomosis may be the only feasible option.^[23,24]^ If complete excision is precluded by proximity to vital structures or extensive involvement, cyst drainage with iodine-tincture cauterization of the lining offers a practical alternative.^[25]^

The incidence of bowel necrosis appears to correlate with both the duration of symptoms at presentation and the degree of intestinal torsion.^[26]^ In our series, only 1 of 21 children who presented within 24 hours exhibited necrosis, whereas 4 of the 7 who sought care after >24 hours had necrotic bowel, highlighting the importance of early recognition and intervention. Among the 7 patients with SBV exceeding 360°, 4 developed intestinal necrosis – significantly more than in those with torsion <360°. Notably, the child with the longest symptom duration showed no necrosis; we speculate that the volvulus was partial and spontaneously detorsed, thereby relieving obstruction. In children with prolonged abdominal pain, meticulous physical examination and appropriate ancillary investigations are therefore essential.

Postoperative recurrence has been widely reported. In Alqahtani review of lymphatic malformations, aspiration and sclerosing-agent injections had the highest recurrence rates, whereas macroscopically complete excision had the lowest.^[27]^ In our study, 26 patients underwent macroscopically complete excision, while 2 patients underwent partial resection with drainage due to extensive mesenteric involvement. No recurrences were detected clinically or radiologically during follow-up.

None of the patients in our series underwent laparoscopic derotation and decompression. Only a few reports describe laparoscopic management of SBV.^[28,29]^ Laparoscopy offers the advantages of minimal invasiveness, faster recovery, and valuable diagnostic information when the clinical picture is unclear. This approach is further supported by Noviello et al.^[30]^ Consequently, laparoscopic surgery should be considered more frequently in patients suspected of having SBV.

This study has several limitations. First, as a retrospective, single-institution analysis, our findings may be subject to selection bias and practices specific to our center. Second, the socioeconomic context of our cohort presents unique challenges: many patients were from low-income rural areas in Southwest China, where a high proportion of “left-behind children” (those raised by grandparents) experienced delayed healthcare-seeking behavior. Although clinically relevant, this demographic factor may restrict the generalizability of our results to populations with different patterns of healthcare access. Third, although our series represents one of the largest single-center experiences with mesenteric lymphangioma-induced volvulus, the absolute sample size remains small because of the condition’s rarity.^[31–35]^ Multicenter studies that include diverse socioeconomic settings are needed to better characterize this pediatric surgical emergency.

## 5. Conclusion

Volvulus secondary to mesenteric lymphangioma is a rare abdominal condition that presents significant diagnostic challenges because of its nonspecific clinical presentation and low incidence. Clinicians should recognize its distinguishing features to enable timely diagnosis and appropriate management. Our findings highlight the importance of including mesenteric lymphangioma-associated volvulus in the differential diagnosis of acute abdominal pain in children.

## Author contributions

**Conceptualization:** Chao Yang.

**Data curation:** Jian Sun.

**Supervision:** Chao Yang.

**Writing – original draft:** Jian Sun.

**Writing – review & editing:** Chao Yang.

## References

[1] Levy AD Cantisani V Miettinen M. Abdominal lymphangiomas: imaging features with pathologic correlation. AJR Am J Roentgenol. 2004;182:1485–91. 15149994 10.2214/ajr.182.6.1821485

[2] Lim DR Kuk JC Kim T Shin EJ. Surgery of multiple lymphangioma in small bowel: a rare case report of chronic gastrointestinal bleeding. Ann Surg Treat Res. 2018;94:52–6. 29333427 10.4174/astr.2018.94.1.52 PMC5765279

[3] Gunadi Kashogi G Prasetya D Fauzi AR Daryanto E Dwihantoro A. Pediatric patients with mesenteric cystic lymphangioma: a case series. Int J Surg Case Rep. 2019;64:89–93. 31622933 10.1016/j.ijscr.2019.09.034 PMC6796738

[4] Protopapas A Papadopoulos D Rodolakis A Markaki S Antsaklis A. Mesenteric lymphangioma presenting as adnexal torsion: case report and literature review. J Clin Ultrasound. 2005;33:90–3. 15674830 10.1002/jcu.20094

[5] Méndez-Gallart R Bautista A Estévez E Rodríguez-Barca P. Abdominal cystic lymphangiomas in pediatrics: surgical approach and outcomes. Acta Chir Belg. 2011;111:374–7. 22299324 10.1080/00015458.2011.11680776

[6] Weeda VB Booij KA Aronson DC. Mesenteric cystic lymphangioma: a congenital and an acquired anomaly? Two cases and a review of the literature. J Pediatr Surg. 2008;43:1206–8. 18558209 10.1016/j.jpedsurg.2008.01.075

[7] Traubici J Daneman A Wales P Gibbs D Fecteau A Kim P. Mesenteric lymphatic malformation associated with small-bowel volvulus – two cases and a review of the literature. Pediatr Radiol. 2002;32:362–5. 11956726 10.1007/s00247-002-0658-y

[8] Yoon HK Han BK. Chronic midgut volvulus with mesenteric lymphangioma: a case report. Pediatr Radiol. 1998;28:611. 9716635 10.1007/s002470050429

[9] Jang JH Lee SL Ku YM An CH Chang ED. Small bowel volvulus induced by mesenteric lymphangioma in an adult: a case report. Korean J Radiol. 2009;10:319–22. 19412523 10.3348/kjr.2009.10.3.319 PMC2672190

[10] Abdulraheem AK Al Sharie AH Al Shalakhti MH Alayoub SY Al-Domaidat HM El-Qawasmeh AE. Mesenteric cystic lymphangioma: a case report. Int J Surg Case Rep. 2021;80:105659. 33636409 10.1016/j.ijscr.2021.105659 PMC7918257

[11] Yang C Qiu T Yang M. Clinical characteristics and risk factors for acute abdomen in patients with abdominal lymphatic malformations. J Vasc Surg Venous Lymphat Disord. 2025;13:101969. 39305949 10.1016/j.jvsv.2024.101969 PMC11764771

[12] Faouzi N Yosra BA Said J. Intestinal volvulus: aetiology, morbidity and mortality in Tunisian children. Afr J Paediatr Surg. 2011;8:147–50. 22005353 10.4103/0189-6725.86050

[13] Akwei S Bhardwaj N Murphy PD. Benign mesenteric lymphangioma presenting as acute pancreatitis: a case report. Cases J. 2009;2:9328. 20062588 10.1186/1757-1626-2-9328 PMC2803988

[14] Siveke JT Braun GS. Small bowel and cecal volvulus due to mesenteric torsion. J Emerg Med. 2004;26:237–9. 14980357 10.1016/j.jemermed.2003.09.007

[15] Singh D Chawla A. The “abdominal whirlpool” sign. Abdom Radiol (NY). 2016;41:1437–8. 26920004 10.1007/s00261-016-0688-9

[16] Patino MO Munden MM. Utility of the sonographic whirlpool sign in diagnosing midgut volvulus in patients with atypical clinical presentations. J Ultrasound Med. 2004;23:397–401. 15055787 10.7863/jum.2004.23.3.397

[17] Chao HC Kong MS Chen JY Lin SJ Lin JN. Sonographic features related to volvulus in neonatal intestinal malrotation. J Ultrasound Med. 2000;19:371–6. 10841057 10.7863/jum.2000.19.6.371

[18] Zalcman M Sy M Donckier V Closset J Gansbeke DV. Helical CT signs in the diagnosis of intestinal ischemia in small-bowel obstruction. AJR Am J Roentgenol. 2000;175:1601–7. 11090385 10.2214/ajr.175.6.1751601

[19] Ding B Wu D Chen S Huang Z. A case report of endoscopic mucosal resection for treating a cystic lymphangioma of the descending colon (in Chinese). J Colorectal Anal Surg. 2023;29:380–4.

[20] Kerkeni Y Zouaoui A Thamri F Boujelbene N Jouini R. Cystic lymphangioma of the small bowel mesentery in a child. J Pediatr. 2022;244:248–9. 34953815 10.1016/j.jpeds.2021.12.032

[21] Rosenblat JM Rozenblit AM Wolf EL DuBrow RA Den EI Levsky JM. Findings of cecal volvulus at CT. Radiology. 2010;256:169–75. 20574094 10.1148/radiol.10092112

[22] Dane B Hindman N Johnson E Rosenkrantz AB. Utility of CT findings in the diagnosis of cecal volvulus. AJR Am J Roentgenol. 2017;209:762–6. 28777650 10.2214/AJR.16.17715

[23] Makni A Chebbi F Fetirich F. Surgical management of intra-abdominal cystic lymphangioma. Report of 20 cases. World J Surg. 2012;36:1037–43. 22358782 10.1007/s00268-012-1515-2

[24] Mhand M Rhoul C Bouhout T Serji B. Cystic lymphangioma of the mesentery in an adult: a case report and literature review. Cureus. 2024;16:e63412. 39070310 10.7759/cureus.63412 PMC11283881

[25] Zhou F Huang H Wang C Ruan X Liu N Han S. Clinical features, diagnosis and treatment of abdominal lymphangioma. Chin J Gen Surg. 2018;33:369–71 in Chinese.

[26] Gui LL Yu L Lu W Xing FZ Xiao BD. Analysis of related factors of congenital intestinal rotation failure with gastric perforation or intestinal necrosis (in Chinese). J Clin Pediatr Surg. 2022;21:69–73.

[27] Alqahtani A Nguyen LT Flageole H Shaw K Laberge JM. 25 years’ experience with lymphangiomas in children. J Pediatr Surg. 1999;34:1164–8. 10442614 10.1016/s0022-3468(99)90590-0

[28] Shimizu S Hara H Muto Y Kido T Miyata R. Successful preoperative diagnosis and laparoscopic management of primary small bowel volvulus: a case report and literature review. Medicine (Baltimore). 2024;103:e39391. 39151494 10.1097/MD.0000000000039391 PMC11332699

[29] Kim KH Kim MC Kim SH Park KJ Jung GJ. Laparoscopic management of a primary small bowel volvulus: a case report. Surg Laparosc Endosc Percutan Tech. 2007;17:335–8. 17710063 10.1097/SLE.0b013e31806c7d04

[30] Noviello C Papparella A Bertozzi M. Abdominal lymphatic malformations in children: case series. Pediatr Med Chir. 2025;47:10. 10.4081/pmc.2025.348 39850011

[31] Mojahid A Benramdane H Mahi NE. Small bowel volvulus induced by cystic lymphangioma in a 12-year-old child: case report. Radiol Case Rep. 2025;20:4381–4. 40583988 10.1016/j.radcr.2025.05.053 PMC12205734

[32] Thapa S Sharma A Upreti D. A huge mesenteric lymphangioma presenting as a small bowel volvulus in a paediatric patient: a case report. Case Rep Pathol. 2022;2022:3033705. 35620582 10.1155/2022/3033705 PMC9130006

[33] Alfadhel SF Alghamdi AA Alzahrani SA. Ileal volvulus secondary to cystic lymphangioma: a rare case report with a literature review. Avicenna J Med. 2019;9:82–5. 31143702 10.4103/ajm.AJM_203_18 PMC6530267

[34] Otsubo Y Ichikawa G Yoshihara S. Small-bowel volvulus caused by mesenteric lymphangioma. Clin Gastroenterol Hepatol. 2020;18:e102. 31077830 10.1016/j.cgh.2019.04.074

[35] Coulibaly Y Keita S Doumbia A Togo A. Volvulus of the jejunum on cystic lymphangioma: about a clinical case. Afr J Paediatr Surg. 2016;13:95–7. 27251660 10.4103/0189-6725.182564 PMC4955446

